# Analysis of the immune response of human dendritic cells to *Mycobacterium tuberculosis* by quantitative proteomics

**DOI:** 10.1186/s12953-016-0095-8

**Published:** 2016-03-08

**Authors:** Chiu-Ping Kuo, Kuo-Song Chang, Jue-Liang Hsu, I-Fang Tsai, Andrew Boyd Lin, Tsai-Yin Wei, Chien-Liang Wu, Yen-Ta Lu

**Affiliations:** Division of Chest Medicine, Department of Internal Medicine, Mackay Memorial Hospital, 92, Sec 2, Chungshan North Road, Taipei, Taiwan; Department of Emergency Medicine, Mackay Memorial Hospital, Taipei, Taiwan; Department of Medical Research, Mackay Memorial Hospital, Taipei, Taiwan; Biology Department, Case Western Reserve University, Cleveland, OH USA; Mackay Junior College of Medicine, Nursing, and Management, Taipei, Taiwan; Department of Medicine, Mackay Medical College, New Taipei City, Taiwan; Graduate Institute of Biotechnology, National Pingtung University of Science and Technology, Pingtung, 91201 Taiwan

**Keywords:** CD13, *M. tuberculosis*, Membrane proteomics, Antigen presentation

## Abstract

**Background:**

The cellular immune response for *Mycobacterium tuberculosis* (*M. tuberculosis*) infection remained incompletely understood. To uncover membrane proteins involved in this infection mechanism, an integrated approach consisting of an organic solvent-assisted membrane protein digestion, stable-isotope dimethyl labeling and liquid chromatography-tandem mass spectrometry (LC-MS/MS) analysis was used to comparatively profile the membrane protein expression of human dendritic cells upon heat-killed *M. tuberculosis* (HKTB) treatment.

**Results:**

Organic solvent-assisted trypsin digestion coupled with stable-isotope labeling and LC-MS/MS analysis was applied to quantitatively analyze the membrane protein expression of THP-1 derived dendritic cells. We evaluated proteins that were upregulated in response to HKTB treatment, and applied STRING website database to analyze the correlations between these proteins. Of the investigated proteins, aminopeptidase N (CD13) was found to be largely expressed after HKTB treatment.

By using confocal microscopy and flow cytometry, we found that membranous CD13 expression was upregulated and was capable of binding to live mycobacteria. Treatment dendritic cell with anti-CD13 antibody during *M. tuberculosis* infection enhanced the ability of T cell activation.

**Conclusions:**

Via proteomics data and STRING analysis, we demonstrated that the highly-expressed CD13 is also associated with proteins involved in the antigen presenting process, especially with CD1 proteins. Increasing expression of CD13 on dendritic cells while *M. tuberculosis* infection and enhancement of T cell activation after CD13 treated with anti-CD13 antibody indicates CD13 positively involved in the pathogenesis of *M. tuberculosis.*

**Electronic supplementary material:**

The online version of this article (doi:10.1186/s12953-016-0095-8) contains supplementary material, which is available to authorized users.

## Background

Despite many effective treatments are available, *M. tuberculosis* remains one of the most successful pathogens on the planet, estimated to have infected nearly one-third of the human population and cause approximately 1.7 million deaths each year [1]. *M. tuberculosis* is typically transmitted via the inhalation of aerosol droplets containing the pathogen. Once inhaled, these small droplets can spread into distal lung alveoli, where they are phagocytosed by alveolar macrophages [2]. Once inside the macrophage, *M. tuberculosis* prevents its phagosome from fusing with digestive lysosomes [3], allowing the pathogen to lay dormant within its host.

While macrophages are the primary targets of the mycobacteria, *M. tuberculosis*’s ability to exist as latent infection suggests that it is also able to suppress other immune responses. Following the initial macrophage response is an acute inflammatory response, causing a rapid recruitment of dendritic cells (DCs) into the airway epithelium [4]. Normally, DCs capture the bacteria, process them, and present their antigens on their cell surfaces to various cells of the immune system. However, it is suggested that specific functions of DCs may be modulated by the mycobacteria*.* More specifically, *M. tuberculosis* has been show to infect DCs and disrupt their capacity to activate and induce primary immune responses in resting naïve T lymphocytes [5–7].

While *M. tuberculosis* infection of macrophages has been studied extensively, little is known about the mechanisms that the mycobacterium uses to mediate cell entry into human DCs. It is plausible that many host factors with important functions and potential therapeutic value have not yet been evaluated. Thus, a global analysis of membrane protein expression in human DCs treated with *M. tuberculosis* could potentially provide further information about the pathogenic mechanisms of tuberculosis. Unfortunately, it is challenging to run a large-scale identification and quantitation of membrane proteins, specifically due to their hydrophobic natures that retard both solubilization in aqueous buffers and downstream enzymatic digestion in a regular bottom-up protein identification pipeline [8, 9]. Recently, possible solutions including formic acid-CNBr/trypsin [10]、high pH/proteinase K [11]、detergent-assisted approach [12]、organic solvent-assisted digestion [13] and tube-gel assisted approach [14, 15] have been used in large-scale membrane proteome studies. Among these methods, the 60 % methanol-assisted trypsin digestion is relatively simple, and the use of a methanol-based buffer circumvents the need for reagents that interfere with chromatographic separation and ionization of the peptides (e.g., detergents, chaotropes, nonvolatile salts). For quantitative aspects, isotope-coded affinity tag [16], isotope coded protein labeling [17], ^18^O labeling [18], stable isotope dimethyl labeling [19], stable isotope labeling by amino acids in cell culture [20] and isobaric tags for relative and absolute quantitation [15, 21] have been introduced for use in comparative membrane proteomics as well as in identification of membrane proteins. Due to its simplicity, effectiveness, and—most importantly—organic solvent compatibility, dimethyl labeling can be efficiently used with 60 % methanol-assisted trypsin digestion of membrane proteins [18]. Therefore, in this study, 60 % methanol-assisted trypsin digestion coupled with this stable-isotope labeling and LC-MS/MS analysis were applied to quantitatively analyze membrane protein expression in THP-1–derived DCs, professional antigen-presenting cells that link the innate and adaptive immunities.

After evaluating proteins that were upregulated in response to heat-killed *M. tuberculosis* treatment, the STRING (Search Tool for the Retrieval of Interacting Genes/Proteins) website database was utilized to analyze associations between these proteins. Of the investigated proteins, aminopeptidase N (CD13) was found to be largely expressed after HKTB treatment. CD13 is a peptidase that affects T cell response by mediating the trimming of major histocompatibility complex (MHC) class II peptides [22], and is also an adhesion molecule involved in leukocyte transendothelial migration into inflammatory sites [23]. In addition, CD13 is involved in phagocytic processes in macrophages and DCs [24]. Recently we have reported that *M. tuberculosis* utilizes CD13 as a mediator of cell entry in human monocytes and macrophages [25]. However, little is known about CD13’s role in mycobacterial interactions with dendritic cells. The results of our study suggest that besides its known functions, CD13 is used by *M. tuberculosis* as an important mediator of cell entry in human dendritic cells to impair the antigen-presenting process and prevent T cell activation.

## Results

### Proteomic profiling of membrane proteins from THP-1-derived DCs treated with or without HKTB

To uncover membrane proteins involved in *M. tuberculosis* infection, an integrated approach consisting of 60 % methanol-assisted membrane protein digestion, stable-isotope dimethyl labeling, LC-MS/MS analysis and database searching was used to comparatively profile the membrane protein expression of THP-1-derived DCs treated with or without HKTB. The membrane proteins of THP-1-derived DCs were extracted by the use of ReadyPrep Membrane II Protein Extraction kit according to the manufacturer’s instructions. A total of 184 proteins derived from the membrane fraction of THP-1-derived DCs treated with or without HKTB were identified through this integrated approach (MascotProtein score and numbers of matched peptide were included in Additional file 1: Table S1). About 62.5 % of proteins were identified as membrane proteins from plasma, endoplasmic reticulum/Golgi, mitochondria, microsome, peroxisome, endosome and lysosome membranes according to their subcellular location shown in UniProt database. Among 49 membrane proteins (around 27 % of total proteins), which were identified as surface membrane proteins, 34 proteins were detected with expression ratios more than 2 and one protein with expression ratios less than 1 when their relative abundance was compared in treated and non-treated samples (Table 1). The ratio distributions of most identified proteins were ~2. These ratios did not converge well into a single value, which may be due to the variance of membrane protein extraction between treated and non-treated samples. Without a reliable internal standard in these identified proteins, a statistic approach was used to discriminate proteins with up or down regulations. We used the average ratio of the membrane proteins (2.48) and standard derivation of these ratios (1.41) to roughly distinguish proteins with significant expression.

**Table 1:** Identified major proteins increased/decreased in response to HKTB stimulation

No.	Name	Protein description	Ratio
1	GGT5	Gamma-glutamyltransferase 5 precursor	8.7
2	ANPEP	Aminopeptidase N, CD13	5.2
3	B*15	HLA class I histocompatibility antigen, B-15 alpha chain precursor	4.6
4	SEL1L	Sel-1 homolog precursor	3.7
5	SLC3A2	4F2 cell-surface antigen heavy chain	3.5
6	VAPA	Vesicle-associated membrane protein-associated protein A	3.4
7	CD74	HLA class II histocompatibility antigen gamma chain	3.4
8	C20orf3	Adipocyte plasma membrane-associated protein	3.4
9	ITGAL	Integrin alpha-L precursor	3.3
10	SLC2A5	Solute carrier family 2, facilitated glucose transporter member 5	3.3
11	GGT1	Gamma-glutamyltranspeptidase 1 precursor	3.2
12	ATP1B1	Sodium/potassium-transporting ATPase subunit beta-1	3.1
13	ITGAX	Integrin alpha-X precursor	3.0
14	TM9SF4	Transmembrane 9 superfamily protein member 4	3.0
15	CPM	Carboxypeptidase M precursor	2.9
16	CD1A	T-cell surface glycoprotein CD1a precursor (CD1a antigen)	2.9
17	PTPRC	Leukocyte common antigen precursor	2.9
18	SLC1A5	Neutral amino acid transporter B(0) (ATB(0))	2.7
19	RAB10	Ras-related protein Rab-10	2.6
20	RAP2A	Ras-related protein Rap-2b precursor	2.6
21	TCIRG1	Vacuolar proton translocating ATPase 116 kDa subunit a isoform 3	2.6
22	KIAA0090	Protein KIAA0090 precursor -	2.5
23	TBXAS1	Thromboxane-A synthase (Cytochrome P450 5A1)	2.5
24	ATP1A1	Sodium/potassium-transporting ATPase alpha-1 chain precursor	2.5
25	CD1C	T-cell surface glycoprotein CD1c precursor (CD1c antigen)	2.5
26	HLA-DR15	HLA class II histocompatibility antigen, DRB1-1 beta chain precursor	2.4
27	TECR	Synaptic glycoprotein SC2	2.4
28	CCDC47	Coiled-coil domain-containing protein 47 precursor	2.3
29	RAB5A	Ras-related protein Rab-5A	2.3
30	ITGB2	Integrin beta-2 precursor (Cell surface adhesion glycoproteins LFA-1/CR3/subunit beta)	2.2
31	ATP6V1B1	Vacuolar ATP synthase subunit B, kidney isoform	2.2
32	EFR3A	Protein KIAA0143 (Fragment)	2.1
33	ESYT1	Protein FAM62A (Membrane-bound C2 domain-containing protein)	2.0
34	SCAMP2	Secretory carrier-associated membrane protein 2	2.0
35	CAP1	Adenylyl cyclase-associated protein 1	0.82

**Fig. 1:**
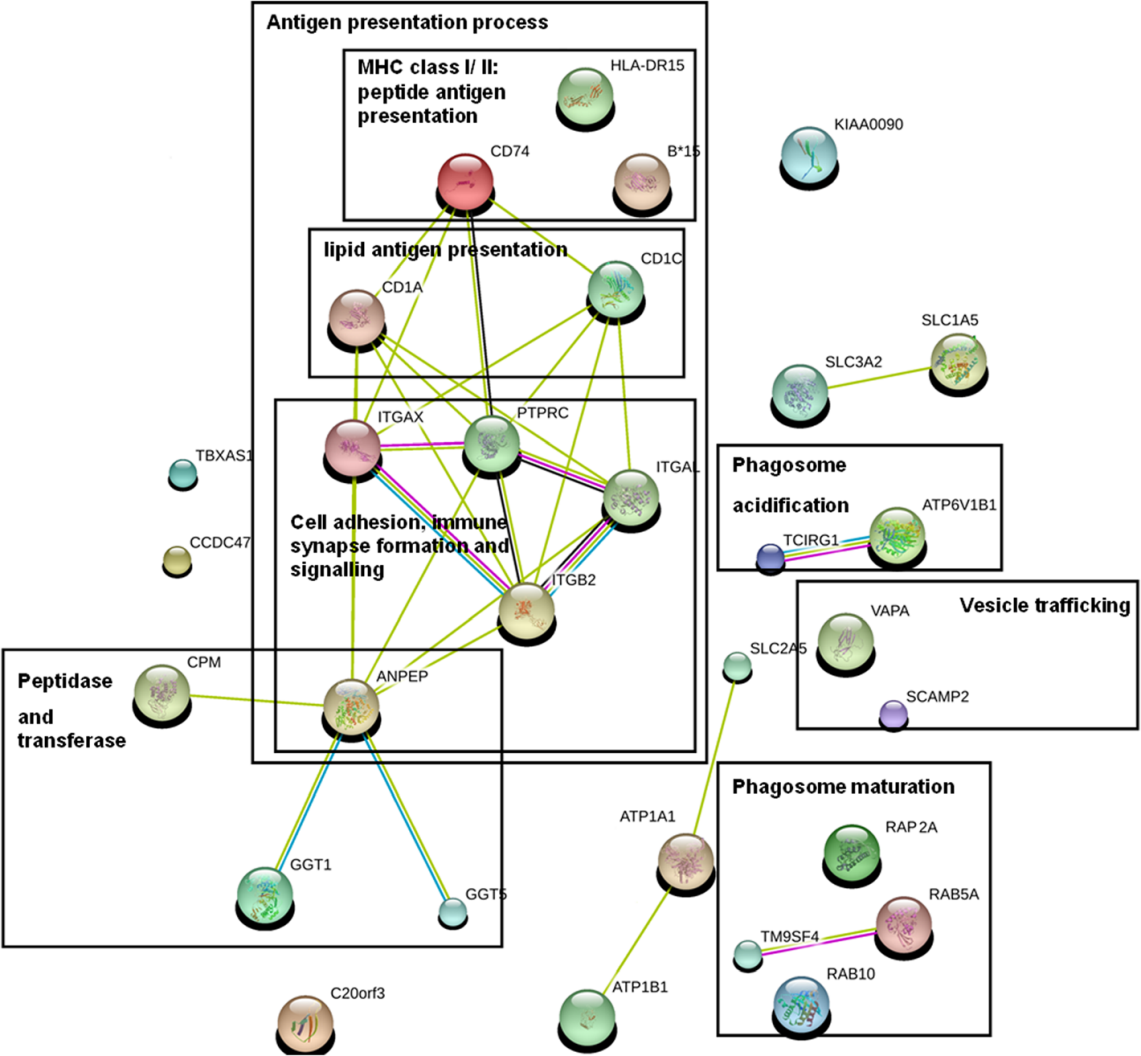
The correlations of these over-expressed membrane proteins. The 34 membrane proteins were analyzed using the open source database and network program STRING, resulting in networks summarized in this figure. Proteins were grouped according to their reported functions, included four clusters (antigen presentation process, peptidase and transferase, vesicle trafficking and phagosome acidification/maturation). Analysis of these networks revealed ANPEP (aminopeptidase, CD13) associated with proteins involved in antigen presentation

To further investigate the role of those up-regulated proteins in mycobacterial infection, STRING database with known and predicted protein-protein interactions was used for functional clustering. The correlations of these over-expressed membrane proteins were shown in Fig. 1. Several functional clusters including proteins involved in extracellular peptide breakdown, antigen presentation, phagosome maturation and markers of dendritic cells were derived from the STRING database. Among these proteins, GGT5 and CD13 are the top two proteins with highest expression ratios (8.7 and 5.2, respectively). To determine which protein would be much relevant in response to clinical tuberculosis, we analyze the expression of GGT5 and CD13 in circulating monocytes from patients with active tuberculosis. The trend of CD13 expression in circulating monocytes from patients with active tuberculosis is also increased as seen in the proteomic analysis. By contrast, the expression of GGT5 in circulating monocytes from patients with active tuberculosis was decreased, which was not compatible to the data seen in proteomic analysis (unpublished observations). Due to its high expression ratio in both in-vitro and clinical samples and potential immune modulatory functions, CD13 was chosen for further study.

**Fig. 2:**
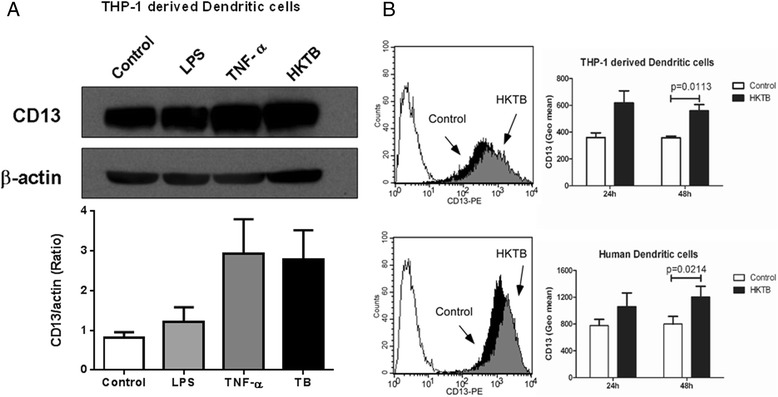
CD13 expression is enhanced on THP-1 derived DCs and human DCs as stimulated with HKTB. **a** Western blotting showing the changes in CD13 level in THP-1 derived DCs treated with LPS and TNF-alpha and HKTB with respect to medium-treated control cells. Protein expressions were quantified by densitometric analysis and CD13 were normalized to the beta-actin of each sample. These experiments were each conducted three times and the results are shown. **b** Flow cytometry analysis of THP-1 derived DCs, and human DCs stained for CD13 antibody and appropriate isotype controls (open histograms). The filled black and gray histograms represent CD13 expression of cells with or without HKTB treatment, respectively. The fluorescence intensity of CD13 was shown as means ± SEM (*n* = 3)

The expression of CD13 was confirmed by Western blotting as shown in Fig. 2a. For THP-1-derived DCs, HKTB promoted the expression of CD 13 as compared to control (HKTB/ control ratio =3.28± 0.44 ), even higher than those by inflammatory stimuli, such as lipopolysaccharide (LPS) and tumor necrosis factor alpha (TNF-α). Furthermore, THP-1-derived DCs and human monocyte-derived DCs were treated with HKTB for 48 h, and surface CD13 expression was evaluated by flow cytometry (Fig. 2b). For THP-1-derived DCs, HKTB significantly increased the expression of CD13 (ratio = 1.56 ± 0.05, *P* = 0.0113). For human DCs, driven from freshly isolated PBMC, HKTB also significantly increased the expression of CD13 (ratio = 1.52 ± 0.07, *P* = 0.0214).

### CD13 mediated the binding and entry of live *M. tuberculosis* onto human DCs

**Fig. 3:**
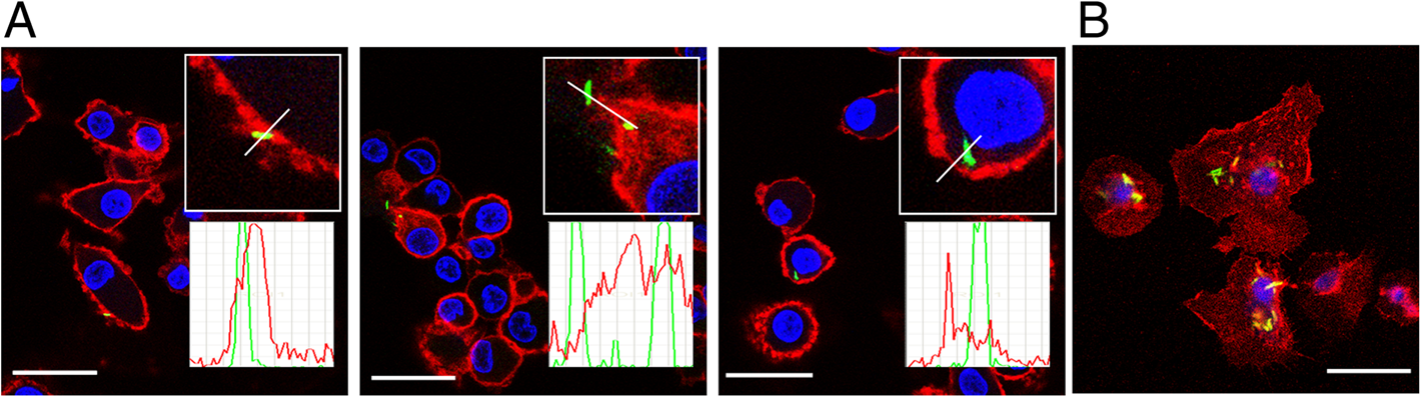
CD13 mediated the binding and entry of live *M. tuberculosis* onto human DCs. **a** The course of *M. tuberculosis* binding to the CD13 and entering into dendritic cells was shown by a series of representative cells. Confocal microscopy showing CD13 on dendritic cells surface stained with anti-CD13 antibody (*red*); *M. tuberculosis* stained with Auramin-Rhodamin T (*green*); cell nucleus stained with DAPI (*blue*); colocalization of *M. tuberculosis* with membrane-bound CD13 on dendritic cells (*yellow*). The profile quantification tool provided by the Leica confocal software was used to obtain graphs with the profiles of intensities of CD13 and *M. tuberculosis* along lineal regions of interest (ROI). **b** At 60 min of incubation, most *M. tuberculosis* was found inside the DCs. Scale bar, 25 μm

To assess whether *M. tuberculosis* interacts with CD13, the binding and entry of live *M. tuberculosis* through surface CD13 of human DCs were examined by confocal microscopy at different time points. Figure 3 shows the results that were obtained in a representative experiment. As *M. tuberculosis* infected human DCs (multiplicity of infection (MOI) = 10) at an indicated time, the samples were fixed by formalin addition to stop the interaction between cells and bacteria. The slides were stained and mounted for subsequent analysis of the mycobacteria-binding process through surface CD13 by confocal microscopy. In the beginning, most mycobacteria were found in the extracellular region and few of them were partially attached to cell surface (Fig. 3a, left panel). As the incubation period increased, *M. tuberculosis* were colocalized with the surface CD13 and internalized into the DC cytoplasm (Fig. 3a, middle panel). After that, most mycobacteria were found inside the DCs (Fig. 3a, right panel). The picture inside each panel shows a drawing of partial enlargement observed in confocal microscopic analysis. The lines traced on the interface of DCs/*M. tuberculosis* depicts the lineal regions of interest that were analyzed using the Lieca confocal software. As shown in each panel of Fig. 3a, analysis of the profiles of green/red channel of these cells confirmed that mycobacteria attached to the surface CD13 of human DCs and were internalized through the binding of the CD13 protein. Interestingly, we observed some mycobacteria still co-localized with CD13 in the cell cytoplasm after 60 min incubation whereas some mycobacteria escaped from the endocytic vesicles and resided freely (Fig. 3b). In summary, we infer that the membranous CD13 on DCs is capable of binding to live mycobacteria.

### CD13 is involved in the inhibition of CD1 expression and T cell response by HKTB

It has been demonstrated that CD13 is involved in cell-surface antigen processing [22], and the CD13 on DCs are able to selectively and efficiently degrade exogenously provided peptide antigens [26] and further influence the capability of triggering T cells. We also wanted to clarify whether CD13 was involved in the lipid antigen presenting process. For this reason, we further studied the influence of highly expressed CD13 molecules in HKTB-stimulated human DCs on CD1 expression and T cell response.

**Fig. 4:**
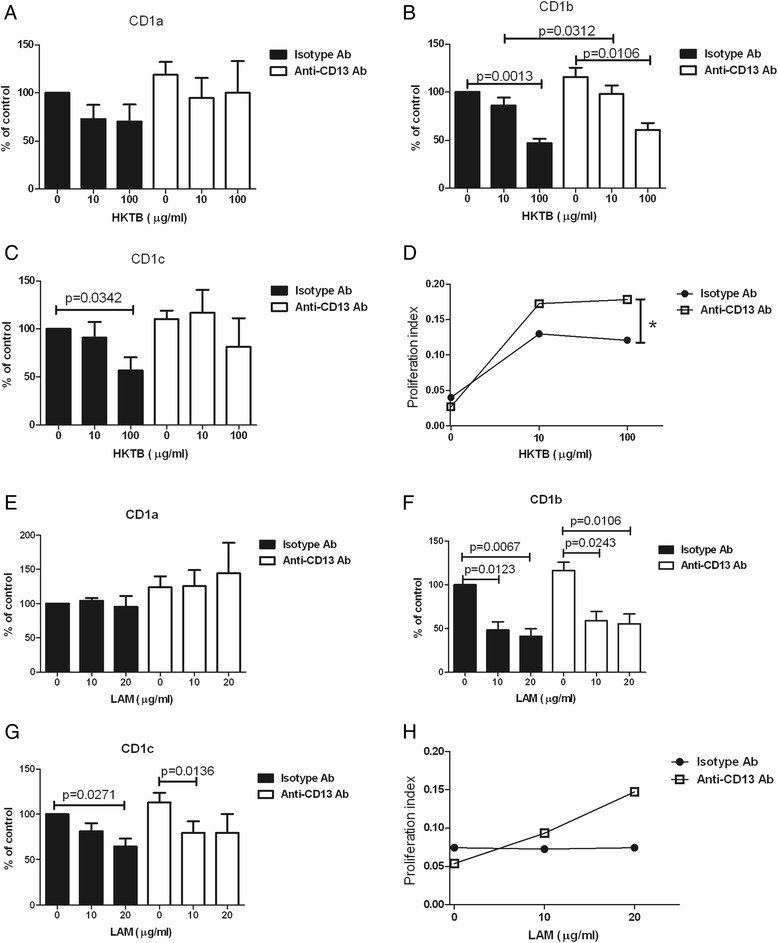
CD13 is involved in the inhibition of CD1 expression and T cell response by HKTB. **a** CD1 expression on dendritic cells infected with or without HKTB for 48 h was measured by flow cytometry. The fluorescence intensity of CD1a was expressed relative to isotype antibody (100 %) and shown as means ± SEM (*n* = 5). The fluorescence intensity of CD1b expression (**b**) and CD1c expression (**c**) were shown as means ± SEM (*n* = 4). **d** CFSE-labeled T cells were cultured with dendritic cells for six days and T cell proliferation was analyzed by flow cytometry. These experiments were conducted with four times and representative result of proliferation index was shown. * *P* <0.05. **e** CD1 expression on dendritic cells infected with or without LAM for 48 h was measured by flow cytometry. The fluorescence intensity of CD1a was expressed relative to isotype antibody (100 %) and shown as means ± SEM (*n* = 5). The fluorescence intensity of CD1b expression (**f**) and CD1c expression (**g**) were shown as means ± SEM (*n* = 4). **h** The results of T cell proliferation were representative of four different experiments

As we used HKTB to stimulate the completely differentiated DC, expressions of CD1b and CD1c decreased. Furthermore, with statistical significance, increasing stimulation with HKTB led to decreasing expressions of CD1 (Fig. 4). We found that HKTB infection of human DCs strongly inhibited CD1b, and, to a lesser extent, CD1c, compared to non-infected DCs with statistical differences (*P* = 0.0013 and *P* = 0.0342, respectively) (Fig. 4a-c). The expression of CD1 represents its non-peptide antigen presentation capability. CD1b and CD1c each present different glycolipids. LAM in the cell wall of *M. tuberculosis* is majorly presented by CD1b [27]. The expression of CD1b and CD1c are affected significantly by stimulation with LAM (*P* = 0.0067 and *P* = 0.0271, respectively) (Fig. 4f-g). We found that the affects of HKTB on CD1c could be reversed by an anti-CD13 antibody (WM15), which also subsequently enhanced the ability of DCs to trigger T cell proliferation (Fig. 4d). We also observed the same trends with LAM treatments (Fig. 4h).

## Discussion

Proteins upregulated in human DCs after HKTB treatment engaged in a diverse range of cellular processes (Fig. 1 and Table 1), suggesting a complex interaction between the bacterium and its host. A proteomic analysis of membrane fraction proteins of THP-1–derived DCs revealed that proteins involved in antigen presentation (HLA class I/II histocompatibility antigens, CD1a, CD1c and CD13) were increased to confront *M. tuberculosis* infection. A cell surface binding protein, CD13 was found to be upregulated 5.2-fold upon HKTB treatment. CD13 is a large cell surface peptidase expressed on the membrane of myeloid dendritic cells [28, 29]. By using Western blot and flow cytometry, we demonstrated that CD13 expression on human DCs was upregulated under inflammatory conditions, especially those induced by HKTB (Fig. 2). However, the physiological relevance of this high expression is not fully understood yet. We have previously reported that CD13 serves as a receptor on monocyte/macrophages that binds to *M. tuberculosis* [25], as well as a possible receptor that mediates of lipid uptake (unpublished observations, [30]). Via proteomics data and STRING analysis, we demonstrated that the highly-expressed CD13 is also associated with proteins involved in the antigen presenting process, especially with CD1 proteins.

CD1 presents glycolipids such as LAM [31], the most immunogenic glycolipid antigen and a key virulence factor on mycobacterial envelopes, to a distinct group of T cells [32]. However, *M. tuberculosis* has been shown to suppress ~60-90 % of CD1a, CD1b, and CD1c expressions on differentiating monocytes [33]. The mycobacterium has also been shown to induce human monocytes to differentiate into CD1-negative DCs, which cannot present lipid antigens to specific T cells [34]. Taken together, these studies suggest that mycobacterial impairment of cellular immune function is strongly associated with CD1 expression [35]. In our study, HKTB and LAM treatments did indeed inhibit the expressions of CD1b and CD1c—but not CD1a—on immature DCs and also decreased T cell proliferation (Fig. 4).

Interestingly, targeting CD13 helped reduce the effects of HKTB and LAM on DCs. Using neutralized antibodies to block the binding receptor CD13 partially reversed the effects of HKTB and LAM treatments on CD1c expression. Because CD1c-positive DCs are an essential group of DCs responsible for naïve T cell proliferation [36], these results suggest that HKTB impairs the ability of DCs to initiate T cell responses. Further testing confirmed that anti-CD13 antibodies also reversed the inhibitive effects of HKTB treatment on T cell proliferation. This was a simpler approach to clarify the interactions between CD13 and CD1, but our results were compatible with STRING database analysis.

Besides revealing information about antigen-presentation, proteomic analysis also revealed that proteins involved in phagosome maturation (Ras-related proteins like Rab5 and Rab 10), phagosome acidification (vacuolar proton ATPases like TCIRG1 and ATP6V1B1) and vesicle trafficking (vesicle-associated/secretory carrier-associated membrane proteins like VAPA and SCAMP2) were upregulated in HKTB-treated DCs. Rab5 and Rab 10, which are both localized to early phagosomes and are required for phagosome maturation, were upregulated by 2.3 fold and 2.6 fold, respectively. Rab10 expression is part of a host cell response during early stages of mycobacterial infection that rescues *Mycobacterium*-containing phagosomes maturation [37, 38]. Meanwhile, vacuolar proton ATPase plays a crucial role in the acidification of phagosomes and is an important initial determinant of mycobacterial Ag85B processing by macrophages [39]. The upregulation of Rab10 and vacuolar proton ATPase could reflect a host anti-bactericidal response to HKTB treatment. We also found that vesicle trafficking-associated proteins VAPA and SCAMP2 were upregulated 3.4 and 2.0-fold, respectively. Unfortunately, the roles of VAPA and SCAMP2 in intracellular transport of membranes are still not clear. Our observation that VAPA and SCAMP2 were upregulated in response to HKTB is a new finding.

Although our STRING analysis didn’t reveal the direct association between CD13 and phagosome acidification-related proteins, CD13 has been reported to not only be a receptor on mycobacteria, but also a mediator that participates in phagosome acidification during *M. tuberculosis* infection [25]. As a result, further analysis is needed to clarify CD13’s interactions with phagosome acidication-related proteins.

Utlimately, we infer that *M. tuberculosis* targets CD13 on dendritic cells to the antigen-presenting process and prevent T cell activation. However, anti-CD13 antibodies were shown to reverse the inhibitive effects of HKTB treatment on T cell proliferation. It has been reported that mycobacteria selectively target surface receptors like dendritic cell-specific intercellular adhesion molecule-3 grabbing nonintegrin (DC-SIGN) [40] to immunosuppress DC function, which prolongs the mycobacteria’s survival. Our results suggest that clinical therapies targeting the CD13 receptor may help reduce *M. tuberculosis’* ability to bind to antigen presenting cells, thereby preventing the inhibition of T cell activation. However, further investigation is still needed on how exactly *M. tuberculosis* enters DCs and inhibits their cell functions through CD13.

## Conclusions

These results add to a growing understanding of the interactions *M. tuberculosis* has with the human immune system. While CD13 is only one of a variety of membrane proteins involved in *M. tuberculosis* infection, evidence provided by this study may lay the groundwork for future tuberculosis target therapies.

## Methods

### Materials

Acetonitrile (ACN), ammonium bicarbonate, 1,4-dithiothreitol (DTT), ethylene glycol, sodium acetate and formaldehyde (37 % solution in H_2_O) were purchased from *J.T. Baker* (NJ, USA). Sodium cyanoborohydride was purchased from Riedel-de Haën (Seelze, Germany). Formic acid (FA), formaldehyde-*D*_2_ (20 % solution in D_2_O), iodoacetamide (IAM), ammonium hydroxide solution (33 %), phorbol-12-myristate-13-acetate (PMA) and dimethylsulfoxide were purchased from Sigma-Aldrich (Germany, Steinheim). Sequencing grade modified trypsin was obtained from Promeg (WI, USA). RPMI 1640 media was purchased from Life Technologies Corporation (Grand Island, NY, USA). ReadyPrep Membrane II Protein Extraction kit was obtained from Bio-Rad (CA, USA). The water used in this study was obtained from Milli-Q® (Millipore) water purification system (Billerica, MA, USA). Lipoarabinomannan (LAM) from *Mycobacterium tuberculosis* Aoyama-B was purchased from Nacalai Tesque (Kyoto, Japan).

#### Ethics statement

This study was conducted according to the principles expressed in the International Conference on Harmonisation (ICH)/WHO Good Clinical Practice standards. Written informed consent was obtained for participation in the study, which was approved by the institutional review board of the Mackay Memorial Hospital.

#### Preparation of THP-1-derived dendritic cells for stimulation with HKTB

The THP-1 cell line (BCRC 60430) was obtained from the Food Industry Research and Development Institute (Hsinchu, Taiwan). THP-1 cells were grown in RPMI 1640 media containing 10 % FCS supplemented with 100 U/ml penicillin and 2 mM L-glutamine. Cells were maintained in a humidified atmosphere with 5 % CO2 at 37 °C. Differentiating THP-1 cells into dendritic cells was achieved by treatment of THP-1 cells with 10 ng/ml PMA, in the presence of 100 ng/ml GM-CSF and 100 ng/ml IL-4 (Peprotech Ltd) [41]. A 10 μg/ml stock solution of PMA was prepared by dissolving PMA in dimethylsulfoxide. HKTB stock solution was prepared by adding 30 ml of RPMI 1640 media into one vial of HKTB powder (100 mg, desiccated *M. tuberculosis*, purchased from BD Difco, GA, USA) and sonicated 5 min for three times. THP-1-derived DCs were then treated with HKTB (10 μg/ml) for three days.

#### Trypsin digestion of membrane proteins

The membrane proteins of THP-1-derived DCs were extracted by the use of ReadyPrep Membrane II Protein Extraction kit (Bio-Rad) according to the manufacturer’s instructions. Briefly, 100-200 mg of wet cell pellets were lysed with lysis buffer. After the removal of insoluble material and unbroken cells, the membrane protein fraction was isolated with ice-cold Na2CO3 followed by centrifugation. Pellets from the control and experimental samples were individually dissolved and digested according to the following procedures. Proteins in the membrane fraction pellet were resuspended in 50 mM ammonium bicarbonate (200 μl, pH 8.3) by vortex and sonication. The proteins were denatured at 95 °C for 5 min then cooled at ice-bath temperature. Due to the hydrophobic nature, the membrane fraction can not be dissolved and digested well by regular digestion protocol. Organic-assisted solubilization and proteolysis were chosen in this study to dissolve and digest membrane proteins. Methanol was added into the protein solution to make a final concentration of 60 % (v/v), facilitating the solubilization of most membrane proteins. Trypsin was then immediately put into this solution in a 1/50 (w/w) trypsin-to-protein ratio followed by incubation for 14 h at 37 °C. The resulting peptide mixtures were lyophilized and stored at -20 °C for further stable-isotope labeling.

#### Stable-isotope dimethyl-labeling of tryptic peptides

The tryptic peptides derived from the control samples which had been dissolved in sodium acetate buffer (100 mM, pH 5-6) were mixed with formaldehyde (4 % in water, 5 μl), vortexed, and then immediately combined with freshly prepared sodium cyanoborohydride (1 M, 5 μl). The mixture was vortexed again and then allowed to react for 5 min. Ammonium hydroxide (4 % in water, 5 μl) was used to consume the excess aldehyde. Deuterium labeling of the experimental samples was performed in a similar manner except using formaldehyde-*d*2 (4 % in water, 5 μl). The tryptic digest derived from the experimental sample was diluted with sodium acetate buffer (100 mM, pH 5-6) and then labeled as described above. The labeled peptides derived from either control or experimental samples were blended and desalted by C18 Easy-Tips (MST, Taipei) for additional fractionation through strong cation exchange (SCX) chromatography.

#### Off-line SCX fractionation of combined mixture

The desalted peptides were redissolved in 0.1 % formic acid and fractionated using a SCX cartridge (5 μm, Vydac, CA, USA). A total of ten fractions eluted with 5, 10, 20, 30, 40, 50, 60, 100, 200, and 500 mM of sodium chloride were serially collected. The resulting fractions were desalted by EasyTipsTM (C SUN, Taipei, Taiwan) and subjected to LC-MS/MS analysis.

#### Mass spectrometric analysis and database searching

The dimethylated tryptic peptides were analyzed using a tandem quadrupole time-of-flight (Q-TOF) mass spectrometer (Micromass, UK) equipped with a nanoflow CapLC system (Waters). The scan range was from m/z 400 to 1600 for MS and m/z 50 to 2000 for MS/MS. For sequencing, the MS/MS spectra were obtained through a survey scan and the automated data-dependent MS analysis was performed by the dynamic exclusion feature built into the MS acquisition software. The MS/MS raw data was processed into a PKL file format using MassLynx 4.0 (Micromass, UK). The resulting PKL file was searched using the Mascot search engine v2.2 (Matrix Science, UK) with the following search parameters: (1) protein database was set to be Swiss-Prot; (2) taxonomy was set as *Homo sapiens* (human); (3) one trypsin missed cleavage was allowed; (4) the precursor and product ion mass tolerance was set at 0.4 Da/0.2 Da; (5) carbamidomethyl (C) was chosen for fixed modification; (6) oxidation (M), deamidated (NQ), Dimethyl (K), Dimethyl (N-term), Dimethyl:2H(4) (K), Dimethyl:2H(4) (N-term) were chosen for variable modifications; (7) proteins with scores above the significance threshold (*p* < 0.05) were shown as significant hits. All MS/MS spectra of identified peptides derived from membrane proteins were further verified by manual interpretation, in particular, using a1 ion in each MS/MS spectrum to verify the N-terminus of the corresponding peptide [42]. The subcellular location and functional annotation of the identified proteins were elucidated by UniProt knowledgebase (Swiss-Prot/TrEMBL) and Gene Ontology Database. The intensity ratios of isotopic isomers were calculated based on peak area or peak intensity from selective ion chromatograms or alternatively, all of the spectra containing both mass peaks of D4- and H4-labeled peptides were combined to produce a composite MS spectrum. The ratios of the D4- and H4-labeled peptides in the composite MS spectra were calculated from the sum of the peak heights of the first three isotopic peaks. Proteins with relative ratio more than 2 were regarded as over-expressed. The accession numbers of these over-expressed surface membrane proteins from HKTB treated THP-1-derived DCs were put together and further correlated by STRING database with known and predicted protein-protein interactions (http://string-db.org/newstring_cgi/show_input_page.pl).

#### Western blotting

THP-1-derived DCs were treated with LPS, TNF-α or HKTB. CD13 protein levels were analyzed by Western blotting to confirm and validate significance of the proteomic findings. Each protein sample was mixed with an electrophoresis buffer containing 2 % SDS and 5 % β-mercaptoethanol and boiled for 10 min. Proteins (5 μg) were separated by electrophoresis on a 10 % SDS-polyacrylamide gel. The fractionated proteins were electroblotted onto a polyvinylidene difluoride membrane. The membranes were blocked at least 2 hrs in 5 % BSA, 0.1 % Tween 20 in TBS (TBST) and then incubated with CD13 antibodies (1:1000 dilution, Abcam, MA, USA), for 1 hr. After washing in TBST, membranes were incubated with peroxidase-conjugated secondary antibodies for 1 hr, and proteins were detected using an enhanced chemiluminescence detection system (PerkinElmer Life and Analytical Sciences, Boston, USA).

#### Human dendritic cells culture

Peripheral blood mononuclear cells (PBMCs) were isolated from the whole blood of healthy adult volunteers by Ficoll-Paque gradient centrifugation. Monocytes were sorted by incubating PBMCs with CD14 microbeads (Miltenyi Biotec) and then the CD14-positive cells were separated by means of a magnetic force. Dendritic cells were generated by culturing monocytes for five days in RPMI-1640 medium with 10 % FBS in the presence of 100 ng/ml GM-CSF and 100 ng/ml IL-4 (Peprotech Ltd). Monocyte-derived DCs were then treated with HKTB solution for two days.

#### Flow cytometry

Cells were stained with PE-conjugated anti-CD13 antibody (clone WM15, BD Pharmingen TM, San Jose, CA, USA). The mean fluorescence intensity of stained cells was measured by FACS Calibur flow cytometry and analyzed by using CellQuest software (BD Bioscience).

##### Live *M tuberculosis* preparation

*M. tuberculosis* were obtained from a clinical virulent strain identified by the Mycobacteriology Laboratory, MacKay Memorial Hospital. Firstly, *M. tuberculosis* was identified by a MPT64 rapid test, arylsulfatase test, and nitrate reductase assay. The identified strain was further confirmed by a commercial TB-PCR kit (COBAS, Taqman MTB test, Roche). Seed stocks of the collected strain were maintained in small aliquots at -80 °C. The *M. tuberculosis* used throughout this study was prepared from the seed stocks by culturing on 7H11 agar plate for 3 weeks at 37 °C in 10 % CO2 humidified atmosphere. The culture medium used was a 7H9 broth supplemented with 0.2 % glycerol, 0.05 % Tween-80 and 10 % oleic acid-albumin-dextrose-catalase enrichment (Difco, Becton Dickinson and Company, MD, USA) to an optical density at 600 nm of 0.3 and used as the inoculum.

#### Confocal microscopy

Dendritic cells were cultured on 18-mm-diameter cover-glass placed in 12-well culture plate and infected with *M. tuberculosis* labeled with Auramin-Rhodamin T. At various time points, unbound bacteria were washed away with PBS and cells were fixed in 4 % formalin. CD13 were stained with Cy-Chrome 5 (Cy5)-conjugated anti-CD13 antibody, and nuclei were stained with 4’-6-Diamidino-2-phenylindole (DAPI). Samples were analyzed by Leica TCS SP5 confocal laser scanning microscopy and quantified by Leica LAS AF software. Use the Leica software profile quantification tool to manually trace a lineal region of interest in each cell that is subjected to analysis. Subsequently, obtain graphs displaying the intensity profiles of CD13/*M. tuberculosis* (green/red channel).

#### T-cell proliferation assay

T cells were purified from CD14-negaitive cells by using the Dynal T cell negative isolation kit (Dynal Biotech Ltd; purity routinely >90 %). T cells were labeled with 5 μM CFSE dye (Invitrogen) for 10 min at 37 degree, washed once, and resuspended in RPMI-1640 medium with 10 % FBS to a final concentration of 2 × 10^6^ cells/ml. CFSE-labebled T cell (2 × 10^5^) were incubated with autologous antigen-loaded dendritic cells (4 × 10^4^) (5 T cells: 1 DC) as previously described [43]. Cells were incubated for 6 days and CFSE dilution was measured by flow cytometry. The proliferation index was calculated by the formula: % of dividing cells/% of non-dividing cells.

#### Statistical analysis

Paired t test was used for analysis. Data are reported as the mean ± SEM. Statistical analysis was performed using Prism 3.0 software (GraphPad Software Inc.). Two-sided tests were used and a P value of < 0.05 was considered statistically significant.

## Additional file

Additional file 1: Table S1. A total of 184 proteins derived from the membrane fraction of THP-1-derived DCs treated with or without HKTB. (DOC 369 kb)

## Abbreviations

DCs: dendritic cells
DC-SIGN: dendritic cell-specific intercellular adhesion molecule-3 grabbing nonintegrin
HKTB: heat-killed *M. tuberculosis*
LAM: Lipoarabinomannan
LC-MS/MS: Liquid chromatography-tandem mass spectrometry
LPS: lipopolysaccharide
STRING: Search Tool for the Retrieval of Interacting Genes/Proteins
TNF-α: tumor necrosis factor alpha
