# Supplementary material for: Analysis of the immune response of human dendritic cells to Mycobacterium tuberculosis by quantitative proteomics
Source: Proteome Sci. 2016 Mar 8;14:5. doi: 10.1186/s12953-016-0095-8 (PMC4782377; doi:10.1186/s12953-016-0095-8)
Supplement: Additional file 1: Table S1. — A total of 184 proteins derived from the membrane fraction of THP-1-derived DCs treated with or without HKTB. (DOC 369 kb) [file 12953_2016_95_MOESM1_ESM.doc]

**Supplemental Table 1: A total of 184 proteins derived from the membrane**

**fraction of THP-1-derived DCs treated with or without HKTB**

| hit | Protein accession | Protein description | MascotProtein score | No. of matched  peptide | Protein expression ratio |
| --- | --- | --- | --- | --- | --- |
| 1 | AMPN_HUMAN | Aminopeptidase N (EC 3.4.11.2) | 581 | 17 | 5.17 |
| 2 | RIB2_HUMAN | Dolichyl-diphosphooligosaccharide--protein glycosyltransferase 63 kDa subunit precursor | 535 | 20 | 3.54 |
| 3 | ITB2_HUMAN | Integrin beta-2 precursor (Cell surface adhesion glycoproteins LFA-1/CR3/p150,95 subunit beta) | 455 | 17 | 2.2 |
| 4 | RIB1_HUMAN | Dolichyl-diphosphooligosaccharide--protein glycosyltransferase 67 kDa subunit precursor | 392 | 17 | 2.44 |
| 5 | ACTB_HUMAN | Actin, cytoplasmic 1 (Beta-actin) | 332 | 15 | 1.24 |
| 6 | TGM2_HUMAN | Protein-glutamine gamma-glutamyltransferase 2 (EC 2.3.2.13) (Tissue transglutaminase) (TGase C) | 268 | 9 | 7.1 |
| 7 | FACE1_HUMAN | CAAX prenyl protease 1 homolog (EC 3.4.24.84) (Prenyl protein-specific endoprotease 1) | 254 | 6 | 2.52 |
| 8 | EF1A1_HUMAN | Elongation factor 1-alpha 1 (EF-1-alpha-1) (Elongation factor 1 A-1) (eEF1A-1) | 230 | 12 | 2.42 |
| 9 | CAP1_HUMAN | Adenylyl cyclase-associated protein 1 (CAP 1) - Homo sapiens (Human) | 208 | 10 | 0.82 |
| 10 | SC22B_HUMAN | Vesicle-trafficking protein SEC22b (SEC22 vesicle-trafficking protein homolog B) | 176 | 6 | 4.42 |
| 11 | OST48_HUMAN | Dolichyl-diphosphooligosaccharide--protein glycosyltransferase 48 kDa subunit precursor | 176 | 8 | 2.31 |
| 12 | CALX_HUMAN | Calnexin precursor (Major histocompatibility complex class I antigen-binding protein p88) (p90) | 173 | 8 | 3.06 |
| 13 | NCPR_HUMAN | NADPH--cytochrome P450 reductase (EC 1.6.2.4) (CPR) (P450R) | 171 | 9 | 3.48 |
| 14 | K2C1_HUMAN | Keratin, type II cytoskeletal 1 (Cytokeratin-1) (CK-1) (Keratin-1) (K1) (67 kDa cytokeratin) | 171 | 13 | 1.02 |
| 15 | GCS1_HUMAN | Mannosyl-oligosaccharide glucosidase (EC 3.2.1.106) (Processing A-glucosidase I) | 168 | 7 | 1.96 |
| 16 | ACTA_HUMAN | Actin, aortic smooth muscle (Alpha-actin-2) | 149 | 8 | 1.24 |
| 17 | MGST3_HUMAN | Microsomal glutathione S-transferase 3 (EC 2.5.1.18) (Microsomal GST-3) (Microsomal GST-III) | 147 | 4 | 1.65 |
| 18 | ITAM_HUMAN | Integrin alpha-M precursor (Cell surface glycoprotein MAC-1 alpha subunit) (CR-3 alpha chain) | 146 | 8 | 1.69 |
| 19 | VDAC1_HUMAN | Voltage-dependent anion-selective channel protein 1 (VDAC-1) (hVDAC1) (Outer mitochondrial membrane | 142 | 7 | 1.99 |
| 20 | ATPA_HUMAN | ATP synthase subunit alpha, mitochondrial precursor (EC 3.6.3.14) | 141 | 4 | 2.61 |
| 21 | RAB10_HUMAN | Ras-related protein Rab-10 | 131 | 4 | 2.63 |
| 22 | STT3A_HUMAN | Dolichyl-diphosphooligosaccharide--protein glycosyltransferase subunit STT3A | 128 | 4 | 1.84 |
| 23 | SURF4_HUMAN | Surfeit locus protein 4 - Homo sapiens (Human) | 126 | 3 | 2.33 |
| 24 | ECHA_HUMAN | Trifunctional enzyme subunit alpha, mitochondrial precursor (TP-alpha) | 120 | 4 | 2.43 |
| 25 | S10AB_HUMAN | Protein S100-A11 (S100 calcium-binding protein A11) (Protein S100C) (Calgizzarin) | 117 | 3 | 2.75 |
| 26 | CY1_HUMAN | Cytochrome c1, heme protein, mitochondrial precursor (Cytochrome c-1) | 115 | 2 | 1.93 |
| 27 | CD45_HUMAN | Leukocyte common antigen precursor (EC 3.1.3.48) (L-CA) (CD45 antigen) (T200) - | 114 | 9 | 2.86 |
| 28 | CBPM_HUMAN | Carboxypeptidase M precursor (EC 3.4.17.12) (CPM) - Homo sapiens (Human) | 113 | 4 | 2.91 |
| 29 | BAP31_HUMAN | B-cell receptor-associated protein 31 (BCR-associated protein Bap31) (p28 Bap31) (Protein CDM) | 108 | 3 | 2.22 |
| 30 | GPNMB_HUMAN | Transmembrane glycoprotein NMB precursor (Transmembrane glycoprotein HGFIN) - Homo sapiens (Human) | 107 | 3 | 1.62 |
| 31 | 1B15_HUMAN | HLA class I histocompatibility antigen, B-15 alpha chain precursor (MHC class I antigen B*15) | 106 | 2 | 4.56 |
| 32 | K22E_HUMAN | Keratin, type II cytoskeletal 2 epidermal (Cytokeratin-2e) (K2e) (CK 2e) - Homo sapiens (Human) | 105 | 4 | 1.06 |
| 33 | APMAP_HUMAN | Adipocyte plasma membrane-associated protein (BSCv protein) - Homo sapiens (Human) | 103 | 4 | 3.37 |
| 34 | AT2A2_HUMAN | Sarcoplasmic/endoplasmic reticulum calcium ATPase 2 (EC 3.6.3.8) (Calcium pump 2) (SERCA2) | 103 | 5 | 2.75 |
| 35 | DHB4_HUMAN | Peroxisomal multifunctional enzyme type 2 (MFE-2) (D-bifunctional protein) (DBP) | 98 | 2 | 2.38 |
| 36 | SPC18_HUMAN | Microsomal signal peptidase 18 kDa subunit (EC 3.4.-.-) (SPase 18 kDa subunit) (SPC18) | 97 | 3 | 2.02 |
| 37 | FA62A_HUMAN | Protein FAM62A (Membrane-bound C2 domain-containing protein) - Homo sapiens (Human) | 96 | 3 | 2.04 |
| 38 | PGH1_HUMAN | Prostaglandin G/H synthase 1 precursor (EC 1.14.99.1) (Cyclooxygenase-1) (COX-1) ( | 96 | 4 | 2.89 |
| 39 | 4F2_HUMAN | 4F2 cell-surface antigen heavy chain (4F2hc) (Lymphocyte activation antigen 4F2 large subunit) | 95 | 10 | 3.2 |
| 40 | ABD12_HUMAN | Abhydrolase domain-containing protein 12 - Homo sapiens (Human) | 94 | 11 | 4.24 |
| 41 | TMCO1_HUMAN | Transmembrane and coiled-coil domain-containing protein 1 (Xenogeneic cross-immune protein PCIA3) - | 92 | 1 | 2.4 |
| 42 | PDIA6_HUMAN | Protein disulfide-isomerase A6 precursor (EC 5.3.4.1) (Protein disulfide isomerase P5) | 90 | 3 | 1.07 |
| 43 | RAB7_HUMAN | Ras-related protein Rab-7 - Homo sapiens (Human) | 87 | 2 | 4.46 |
| 44 | RAP2B_HUMAN | Ras-related protein Rap-2b precursor - Homo sapiens (Human) | 85 | 2 | 2.57 |
| 45 | ABCD3_HUMAN | ATP-binding cassette sub-family D member 3 (70 kDa peroxisomal membrane protein) (PMP70) | 85 | 5 | 2.38 |
| 46 | MTCH2_HUMAN | Mitochondrial carrier homolog 2 (Met-induced mitochondrial protein) - Homo sapiens (Human) | 84 | 3 | 2.35 |
| 47 | GGT5_HUMAN | Gamma-glutamyltransferase 5 precursor (EC 2.3.2.2) (Gamma-glutamyltranspeptidase 5) | 80 | 2 | 8.7 |
| 48 | ADT2_HUMAN | ADP/ATP translocase 2 (Adenine nucleotide translocator 2) (ANT 2) | 80 | 6 | 2.28 |
| 49 | ATPK_HUMAN | ATP synthase f chain, mitochondrial (EC 3.6.3.14) - Homo sapiens (Human) | 79 | 5 | 2.32 |
| 50 | TBAK_HUMAN | Tubulin alpha-ubiquitous chain (Alpha-tubulin ubiquitous) (Tubulin K-alpha-1) - Homo sapiens (Human | 79 | 2 | 1.33 |
| 51 | FRIL_HUMAN | Ferritin light chain (Ferritin L subunit) - Homo sapiens (Human) | 78 | 2 | 0.68 |
| 52 | SSRA_HUMAN | Translocon-associated protein subunit alpha precursor (TRAP-alpha) (Signal sequence receptor subuni | 76 | 1 | 1.8 |
| 53 | ITAL_HUMAN | Integrin alpha-L precursor (Leukocyte adhesion glycoprotein LFA-1 alpha chain) (LFA-1A) (Leukocyte | 75 | 2 | 3.33 |
| 54 | K0090_HUMAN | Protein KIAA0090 precursor - Homo sapiens (Human) | 74 | 1 | 2.53 |
| 55 | OXRP_HUMAN | 150 kDa oxygen-regulated protein precursor (Orp150) (Hypoxia up-regulated 1) - Homo sapiens (Human) | 73 | 1 | 2.44 |
| 56 | NCB5R_HUMAN | NADH-cytochrome b5 reductase (EC 1.6.2.2) (B5R) (Diaphorase-1) (Cytochrome b5 reductase 3) | 73 | 5 | 2.33 |
| 57 | LACTB_HUMAN | Serine beta-lactamase-like protein LACTB - Homo sapiens (Human) | 72 | 3 | 3.1 |
| 58 | ANXA2_HUMAN | Annexin A2 (Annexin II) (Lipocortin II) (Calpactin I heavy chain) (Chromobindin-8) (p36) | 72 | 1 | 2.06 |
| 59 | VDAC3_HUMAN | Voltage-dependent anion-selective channel protein 3 (VDAC-3) (hVDAC3) (Outer mitochondrial membrane | 71 | 4 | 1.81 |
| 60 | PRDX6_HUMAN | Peroxiredoxin-6 (EC 1.11.1.15) (Antioxidant protein 2) (1-Cys peroxiredoxin) (1-Cys PRX) | 71 | 2 | 2.52 |
| 61 | SCAM2_HUMAN | Secretory carrier-associated membrane protein 2 (Secretory carrier membrane protein 2) | 70 | 2 | 2.04 |
| 62 | RAB5A_HUMAN | Ras-related protein Rab-5A - Homo sapiens (Human) | 69 | 2 | 2.26 |
| 63 | CD1C_HUMAN | T-cell surface glycoprotein CD1c precursor (CD1c antigen) - Homo sapiens (Human) | 69 | 2 | 2.5 |
| 64 | DHB12_HUMAN | Estradiol 17-beta-dehydrogenase 12 (EC 1.1.1.62) (17-beta-HSD 12) | 68 | 3 | 2.34 |
| 65 | HYEP_HUMAN | Epoxide hydrolase 1 (EC 3.3.2.9) (Microsomal epoxide hydrolase) (Epoxide hydratase) - Homo sapiens | 68 | 5 | 1.35 |
| 66 | THAS_HUMAN | Thromboxane-A synthase (EC 5.3.99.5) (TXA synthase) (TXS) (Cytochrome P450 5A1) | 67 | 1 | 2.53 |
| 67 | UN93B_HUMAN | UNC93 homolog B1 (UNC-93B protein) (hUNC93B1) - Homo sapiens (Human) | 67 | 1 | 1.71 |
| 68 | AAAT_HUMAN | Neutral amino acid transporter B(0) (ATB(0)) (Sodium-dependent neutral amino acid transporter type | 67 | 2 | 2.72 |
| 69 | LAMP1_HUMAN | Lysosome-associated membrane glycoprotein 1 precursor (LAMP-1) (CD107a antigen) | 66 | 2 | 1.47 |
| 70 | F13A_HUMAN | Coagulation factor XIII A chain precursor (EC 2.3.2.13) (Coagulation factor XIIIa) | 66 | 1 | 0.7 |
| 71 | G6PI_HUMAN | Glucose-6-phosphate isomerase (EC 5.3.1.9) (GPI) (Phosphoglucose isomerase) (PGI) | 65 | 1 | 0.8 |
| 72 | RAP1A_HUMAN | Ras-related protein Rap-1A precursor (GTP-binding protein smg-p21A) (Ras-related protein Krev-1) | 65 | 1 | 1.95 |
| 73 | TCPD_HUMAN | T-complex protein 1 subunit delta (TCP-1-delta) (CCT-delta) (Stimulator of TAR RNA-binding) | 65 | 1 | 2.13 |
| 74 | CKAP4_HUMAN | Cytoskeleton-associated protein 4 (63 kDa membrane protein) (p63) - Homo sapiens (Human) | 65 | 2 | 1.48 |
| 75 | PDIA1_HUMAN | Protein disulfide-isomerase precursor (EC 5.3.4.1) (PDI) (Prolyl 4-hydroxylase subunit beta) | 64 | 1 | 1.08 |
| 76 | CYB5B_HUMAN | Cytochrome b5 type B precursor (Cytochrome b5 outer mitochondrial membrane isoform) - Homo sapiens | 63 | 2 | 2.04 |
| 77 | VAPA_HUMAN | Vesicle-associated membrane protein-associated protein A (VAMP-associated protein A) (VAMP-A) | 62 | 3 | 3.43 |
| 78 | GTR5_HUMAN | Solute carrier family 2, facilitated glucose transporter member 5 | 61 | 1 | 3.27 |
| 79 | ITAX_HUMAN | Integrin alpha-X precursor (Leukocyte adhesion glycoprotein p150,95 alpha chain) | 61 | 2 | 3.01 |
| 80 | ENOA_HUMAN | Alpha-enolase (EC 4.2.1.11) (2-phospho-D-glycerate hydro-lyase) (Non-neural enolase) (NNE) | 60 | 2 | 1.02 |
| 81 | TMEDA_HUMAN | Transmembrane emp24 domain-containing protein 10 precursor (Transmembrane protein Tmp21) | 57 | 6 | 2.04 |
| 82 | K2C3_HUMAN | Keratin, type II cytoskeletal 3 (Cytokeratin-3) (CK-3) (Keratin-3) (K3) (65 kDa cytokeratin) | 57 | 2 | 0.86 |
| 83 | K0143_HUMAN | Protein KIAA0143 (Fragment) - Homo sapiens (Human) | 57 | 2 | 2.05 |
| 84 | 6PGD_HUMAN | 6-phosphogluconate dehydrogenase, decarboxylating (EC 1.1.1.44) - Homo sapiens (Human) | 56 | 2 | 2.78 |
| 85 | TOM22_HUMAN | Mitochondrial import receptor subunit TOM22 homolog | 55 | 2 | 1.69 |
| 86 | API5_HUMAN | Apoptosis inhibitor 5 (API-5) (Fibroblast growth factor 2-interacting factor) (FIF) (Protein XAGL) | 55 | 1 | 2.26 |
| 87 | ATP5H_HUMAN | ATP synthase D chain, mitochondrial (EC 3.6.3.14) - Homo sapiens (Human) | 54 | 2 | 2.07 |
| 88 | MPCP_HUMAN | Phosphate carrier protein, mitochondrial precursor (PTP) (Solute carrier family 25 member 3) | 54 | 2 | 2.38 |
| 89 | SGPL1_HUMAN | Sphingosine-1-phosphate lyase 1 (EC 4.1.2.27) (SP-lyase) (hSPL) (Sphingosine-1-phosphate aldolase) | 54 | 1 | 6.32 |
| 90 | CCD47_HUMAN | Coiled-coil domain-containing protein 47 precursor - Homo sapiens (Human) | 54 | 2 | 2.27 |
| 91 | MGAT1_HUMAN | Alpha-1,3-mannosyl-glycoprotein 2-beta-N-acetylglucosaminyltransferase (EC 2.4.1.101) | 53 | 1 | 1.67 |
| 92 | G3P_HUMAN | Glyceraldehyde-3-phosphate dehydrogenase (EC 1.2.1.12) (GAPDH) - Homo sapiens (Human) | 53 | 1 | 1.73 |
| 93 | PDIA3_HUMAN | Protein disulfide-isomerase A3 precursor (EC 5.3.4.1) (Disulfide isomerase ER-60) (ERp60) | 53 | 1 | 2.49 |
| 94 | VPP3_HUMAN | Vacuolar proton translocating ATPase 116 kDa subunit a isoform 3 (V-ATPase 116 kDa isoform a3) | 52 | 2 | 2.56 |
| 95 | GPDM_HUMAN | Glycerol-3-phosphate dehydrogenase, mitochondrial precursor (EC 1.1.99.5) (GPD-M) (GPDH-M) (mtGPD) | 51 | 2 | 3.45 |
| 96 | CATD_HUMAN | Cathepsin D precursor (EC 3.4.23.5) [Contains: Cathepsin D light chain; Cathepsin D heavy chain] | 49 | 3 | 1.33 |
| 97 | TFR1_HUMAN | Transferrin receptor protein 1 (TfR1) (TR) (TfR) (Trfr) (CD71 antigen) (T9) (p90) - | 49 | 1 | 1.35 |
| 98 | AT1A1_HUMAN | Sodium/potassium-transporting ATPase alpha-1 chain precursor (EC 3.6.3.9) (Sodium pump 1) | 49 | 3 | 2.52 |
| 99 | ABCD1_HUMAN | ATP-binding cassette sub-family D member 1 (Adrenoleukodystrophy protein) (ALDP) - Homo sapiens | 48 | 2 | 3.73 |
| 100 | ITPR2_HUMAN | Inositol 1,4,5-trisphosphate receptor type 2 (Type 2 inositol 1,4,5-trisphosphate receptor) | 47 | 2 | 3.08 |
| 101 | LMAN1_HUMAN | ERGIC-53 protein precursor (ER-Golgi intermediate compartment 53 kDa protein) | 46 | 3 | 2.39 |
| 102 | NICA_HUMAN | Nicastrin precursor - Homo sapiens (Human) | 46 | 1 | 1.7 |
| 103 | SEL1L_HUMAN | Sel-1 homolog precursor (Suppressor of lin-12-like protein) (Sel-1L) - Homo sapiens (Human) | 45 | 1 | 3.71 |
| 104 | VATB1_HUMAN | Vacuolar ATP synthase subunit B, kidney isoform (EC 3.6.3.14) (V-ATPase B1 subunit) | 45 | 1 | 2.19 |
| 105 | GPSN2_HUMAN | Synaptic glycoprotein SC2 - Homo sapiens (Human) | 45 | 1 | 2.36 |
| 106 | LYRIC_HUMAN | Protein LYRIC (Lysine-rich CEACAM1 co-isolated protein) (3D3/lyric) (Metastasis adhesion protein) | 45 | 1 | 1.26 |
| 107 | MIRO2_HUMAN | Mitochondrial Rho GTPase 2 (EC 3.6.5.-) (MIRO-2) (hMiro-2) (Ras homolog gene family member T2) | 44 | 2 | 1.88 |
| 108 | SPFH2_HUMAN | SPFH domain-containing protein 2 precursor - Homo sapiens (Human) | 44 | 2 | 1.75 |
| 109 | UBIQ_HUMAN | Ubiquitin - Homo sapiens (Human) | 44 | 1 | 1.91 |
| 110 | MET7B_HUMAN | Methyltransferase-like protein 7B precursor (EC 2.1.1.-) - Homo sapiens (Human) | 44 | 2 | 0.93 |
| 111 | PRAF3_HUMAN | PRA1 family protein 3 (ARL-6-interacting protein 5) | 44 | 1 | 4.6 |
| 112 | GALT2_HUMAN | Polypeptide N-acetylgalactosaminyltransferase 2 (EC 2.4.1.41) | 44 | 1 | 2.19 |
| 113 | GGT1_HUMAN | Gamma-glutamyltranspeptidase 1 precursor (EC 2.3.2.2) (Gamma-glutamyltransferase 1) (GGT 1) | 42 | 3 | 3.19 |
| 114 | LDHA_HUMAN | L-lactate dehydrogenase A chain (EC 1.1.1.27) (LDH-A) (LDH muscle subunit) (LDH-M) | 41 | 3 | 1.98 |
| 115 | BST1_HUMAN | ADP-ribosyl cyclase 2 precursor (EC 3.2.2.5) (Cyclic ADP-ribose hydrolase 2) (cADPr hydrolase 2) | 41 | 2 | 1.87 |
| 116 | HG2A_HUMAN | HLA class II histocompatibility antigen gamma chain (HLA-DR antigens-associated invariant chain) | 41 | 1 | 3.43 |
| 117 | HXK1_HUMAN | Hexokinase-1 (EC 2.7.1.1) (Hexokinase type I) (HK I) (Brain form hexokinase) - Homo sapiens (Human) | 41 | 3 | 1.5 |
| 118 | LEG9_HUMAN | Galectin-9 (HOM-HD-21) (Ecalectin) - Homo sapiens (Human) | 40 | 1 | 1.83 |
| 119 | SQRD_HUMAN | Sulfide:quinone oxidoreductase, mitochondrial precursor (EC 1.-.-.-) - Homo sapiens (Human) | 40 | 2 | 3.09 |
| 120 | ADPGK_HUMAN | ADP-dependent glucokinase (EC 2.7.1.147) (ADPGK) (ADP-GK) (RbBP-35) - Homo sapiens (Human) | 40 | 1 | 3.1 |
| 121 | VKORL_HUMAN | Vitamin K epoxide reductase complex subunit 1-like protein 1 (VKORC1-like protein 1) - Homo sapiens | 39 | 1 | 1.72 |
| 122 | 2DRA_HUMAN | HLA class II histocompatibility antigen, DR alpha chain precursor (MHC class II antigen DRA) - Homo sapiens (Human) | 39 | 3 | 1.97 |
| 123 | HS90B_HUMAN | Heat shock protein HSP 90-beta (HSP 84) (HSP 90) - Homo sapiens (Human) | 38 | 2 | 0.87 |
| 124 | AT1B1_HUMAN | Sodium/potassium-transporting ATPase subunit beta-1 | 37 | 1 | 3.05 |
| 125 | U373_HUMAN | UPF0373 protein precursor - Homo sapiens (Human) | 36 | 1 | 1.93 |
| 126 | SC23A_HUMAN | Protein transport protein Sec23A (SEC23-related protein A) - Homo sapiens (Human) | 36 | 1 | 3.07 |
| 127 | HSP7C_HUMAN | Heat shock cognate 71 kDa protein (Heat shock 70 kDa protein 8) - Homo sapiens (Human) | 36 | 3 | 1.39 |
| 128 | DERL1_HUMAN | Derlin-1 (Degradation in endoplasmic reticulum protein 1) (Der1-like protein 1) (DERtrin-1) - Homo sapiens (Human) | 36 | 1 | 2.24 |
| 129 | HEXB_HUMAN | Beta-hexosaminidase beta chain precursor (EC 3.2.1.52) (N-acetyl-beta-glucosaminidase) | 36 | 3 | 0.77 |
| 130 | RL17_HUMAN | 60S ribosomal protein L17 (L23) - Homo sapiens (Human) | 36 | 1 | 1.43 |
| 131 | NCLN_HUMAN | Nicalin precursor (Nicastrin-like protein) - Homo sapiens (Human) | 36 | 1 | 2.1 |
| 132 | MYO1F_HUMAN | Myosin If (Myosin-IE) - Homo sapiens (Human) | 35 | 1 | 2.84 |
| 133 | BR44_HUMAN | Brain protein 44 - Homo sapiens (Human) | 35 | 1 | 1.88 |
| 134 | HS90A_HUMAN | Heat shock protein HSP 90-alpha (HSP 86) (NY-REN-38 antigen) - Homo sapiens (Human) | 35 | 3 | 0.84 |
| 135 | SATT_HUMAN | Neutral amino acid transporter A (SATT) (Alanine/serine/cysteine/ threonine transporter) (ASCT1) - | 34 | 2 | 1.83 |
| 136 | K1C10_HUMAN | Keratin, type I cytoskeletal 10 (Cytokeratin-10) (CK-10) (Keratin-10) (K10) - Homo sapiens (Human) | 33 | 2 | 0.84 |
| 137 | SOAT1_HUMAN | Sterol O-acyltransferase 1 (EC 2.3.1.26) (Cholesterol acyltransferase 1) (Acyl coenzyme A:cholester | 32 | 2 | 4.15 |
| 138 | TM9S4_HUMAN | Transmembrane 9 superfamily protein member 4 - Homo sapiens (Human) | 32 | 3 | 2.99 |
| 139 | DHC24_HUMAN | 24-dehydrocholesterol reductase precursor (EC 1.3.1.-) (3-beta-hydroxysterol delta-24-reductase) | 32 | 1 | 1.73 |
| 140 | LAMP2_HUMAN | Lysosome-associated membrane glycoprotein 2 precursor (LAMP-2) (CD107b antigen) - Homo sapiens | 32 | 1 | 1.46 |
| 141 | PHB2_HUMAN | Prohibitin-2 (B-cell receptor-associated protein BAP37) (Repressor of estrogen receptor activity) | 31 | 2 | 2.05 |
| 142 | LMAN2_HUMAN | Vesicular integral-membrane protein VIP36 precursor (GP36b glycoprotein) (Lectin, mannose-binding 2 | 31 | 1 | 1.74 |
| 143 | KCRB_HUMAN | Creatine kinase B-type (EC 2.7.3.2) (Creatine kinase B chain) (B-CK) - Homo sapiens (Human) | 30 | 2 | 1.64 |
| 144 | NPM_HUMAN | Nucleophosmin (NPM) (Nucleolar phosphoprotein B23) (Numatrin) (Nucleolar protein NO38) | 30 | 1 | 1.14 |
| 145 | SFPQ_HUMAN | Splicing factor, proline- and glutamine-rich | 29 | 1 | 1.78 |
| 146 | TOM7_HUMAN | Probable mitochondrial import receptor subunit TOM7 homolog (Translocase of outer membrane 7 kDa su | 29 | 1 | 1.94 |
| 147 | SFXN3_HUMAN | Sideroflexin-3 - Homo sapiens (Human) | 28 | 1 | 2.08 |
| 148 | FDFT_HUMAN | Squalene synthetase (EC 2.5.1.21) (SQS) (SS) (Farnesyl-diphosphate farnesyltransferase) | 28 | 1 | 1.63 |
| 149 | TAGL2_HUMAN | Transgelin-2 (SM22-alpha homolog) - Homo sapiens (Human) | 28 | 1 | 0.61 |
| 150 | PSME2_HUMAN | Proteasome activator complex subunit 2 (Proteasome activator 28-subunit beta) (PA28beta) (PA28b) | 28 | 1 | 4.9 |
| 151 | H2B1B_HUMAN | Histone H2B type 1-B (H2B.f) (H2B/f) (H2B.1) - Homo sapiens (Human) | 28 | 1 | 0.74 |
| 152 | TMED9_HUMAN | Transmembrane emp24 domain-containing protein 9 precursor (Glycoprotein 25L2) - Homo sapiens | 27 | 3 | 2.18 |
| 153 | GPNMB_HUMAN | Transmembrane glycoprotein NMB precursor (Transmembrane glycoprotein HGFIN) - Homo sapiens (Human) | 62 | 1 | 1.8 |
| 154 | SURF4_HUMAN | Surfeit locus protein 4 - Homo sapiens (Human) | 59 | 2 | 2.17 |
| 155 | VPP3_HUMAN | Vacuolar proton translocating ATPase 116 kDa subunit a isoform 3 (V-ATPase 116 kDa isoform a3) | 56 | 2 | 2.54 |
| 156 | ADT2_HUMAN | ADP/ATP translocase 2 (Adenine nucleotide translocator 2) (ANT 2) (ADP,ATP carrier protein 2) | 76 | 2 | 2.4 |
| 157 | BST1_HUMAN | ADP-ribosyl cyclase 2 precursor (EC 3.2.2.5) (Cyclic ADP-ribose hydrolase 2) (cADPr hydrolase 2) | 40 | 1 | 1.87 |
| 158 | 4F2_HUMAN | 4F2 cell-surface antigen heavy chain (4F2hc) (Lymphocyte activation antigen 4F2 large subunit) | 128 | 4 | 3.45 |
| 159 | TBAK_HUMAN | Tubulin alpha-ubiquitous chain (Alpha-tubulin ubiquitous) (Tubulin K-alpha-1) - Homo sapiens | 106 | 2 | 1.33 |
| 160 | MGAT1_HUMAN | Alpha-1,3-mannosyl-glycoprotein 2-beta-N-acetylglucosaminyltransferase (EC 2.4.1.101) | 51 | 1 | 1.67 |
| 161 | ABD12_HUMAN | Abhydrolase domain-containing protein 12 - Homo sapiens (Human) | 46 | 1 | 2.91 |
| 162 | 2B11_HUMAN | HLA class II histocompatibility antigen, DRB1-1 beta chain precursor (MHC class I antigen DRB1*1) | 39 | 1 | 2.43 |
| 163 | VDAC1_HUMAN | Voltage-dependent anion-selective channel protein 1 (VDAC-1) (hVDAC1) (Outer mitochondrial membrane | 51 | 2 | 1.71 |
| 164 | RIB2_HUMAN | Dolichyl-diphosphooligosaccharide--protein glycosyltransferase 63 kDa subunit precursor | 71 | 2 | 4.66 |
| 165 | S10AB_HUMAN | Protein S100-A11 (S100 calcium-binding protein A11) (Protein S100C) (Calgizzarin) (MLN 70) | 73 | 1 | 1.97 |
| 166 | CD1A_HUMAN | T-cell surface glycoprotein CD1a precursor (CD1a antigen) (T-cell surface antigen T6/Leu-6) | 69 |  | 2.89 |
| 167 | ANXA5_HUMAN | Annexin A5 (Annexin V) (Lipocortin V) (Endonexin II) (Calphobindin I) (CBP-I) | 27 | 1 | 1.21 |
| 168 | S17A5_HUMAN | Sialin (Solute carrier family 17 member 5) (Sodium/sialic acid cotransporter) (AST) | 27 | 1 | 2.07 |
| 169 | GNAQ_HUMAN | Guanine nucleotide-binding protein G(q) subunit alpha (Guanine nucleotide-binding protein alpha-q) | 26 | 1 | 2.24 |
| 170 | ARL8B_HUMAN | ADP-ribosylation factor-like protein 8B (ADP-ribosylation factor-like protein 10C) | 25 | 3 | 2.33 |
| 171 | VTNC_HUMAN | Vitronectin precursor (Serum-spreading factor) (S-protein) (V75) [Contains: Vitronectin V65 subunit | 25 | 1 | 2.47 |
| 172 | BAT5_HUMAN | Protein BAT5 (HLA-B-associated transcript 5) (Protein G5) - Homo sapiens (Human) | 25 | 1 | 1.91 |
| 173 | CLH1_HUMAN | Clathrin heavy chain 1 (CLH-17) - Homo sapiens (Human) | 24 | 2 |  |
| 174 | SFRS8_HUMAN | Splicing factor, arginine/serine-rich 8 (Suppressor of white apricot protein homolog) - Homo sapien | 24 | 1 |  |
| 175 | VIME_HUMAN | Vimentin - Homo sapiens (Human) | 23 | 1 |  |
| 176 | IMMT_HUMAN | Mitochondrial inner membrane protein (Mitofilin) (p87/89) (Proliferation-inducing gene 4 protein) - | 23 | 2 |  |
| 177 | PPAP_HUMAN | Prostatic acid phosphatase precursor (EC 3.1.3.2) - Homo sapiens (Human) | 23 | 2 |  |
| 178 | RAN_HUMAN | GTP-binding nuclear protein Ran (GTPase Ran) (Ras-like protein TC4) (Androgen receptor-associated p | 22 | 1 |  |
| 179 | AT131_HUMAN | Probable cation-transporting ATPase 13A1 (EC 3.6.3.-) - Homo sapiens (Human) | 21 | 2 |  |
| 180 | VATL_HUMAN | Vacuolar ATP synthase 16 kDa proteolipid subunit (EC 3.6.3.14) - Homo sapiens (Human) | 20 | 1 |  |
| 180 | VDAC2_HUMAN | Voltage-dependent anion-selective channel protein 2 (VDAC-2) (hVDAC2) (Outer mitochondrial membrane | 20 | 2 |  |
| 181 | MTCH1_HUMAN | Mitochondrial carrier homolog 1 (Presenilin-associated protein) - Homo sapiens (Human) | 20 | 2 |  |
| 182 | 2B14_HUMAN | HLA class II histocompatibility antigen, DRB1-4 beta chain precursor (MHC class I antigen DRB1*4) | 20 | 1 |  |
| 183 | TPP1_HUMAN | Tripeptidyl-peptidase 1 precursor (EC 3.4.14.9) (Tripeptidyl-peptidase I) (TPP-I) | 19 | 2 |  |
| 184 | STT3B_HUMAN | Dolichyl-diphosphooligosaccharide--protein glycosyltransferase subunit STT3B (EC 2.4.1.119) | 18 | 1 |  |
